# Haushaltsklima, Alleinleben und gesundheitsbezogene Lebensqualität während des COVID-19-Lockdowns in Deutschland

**DOI:** 10.1007/s11553-021-00865-6

**Published:** 2021-06-25

**Authors:** Marina Zurek, Luisa Friedmann, Emilia Kempter, André Silva Chaveiro, Adekunle Adedeji, Franka Metzner

**Affiliations:** 1 Wirtschaft & Medien, Hochschule Fresenius für Management, Hamburg, Deutschland; 2 Klinik für Kinder- und Jugendpsychiatrie, -psychotherapie und -psychosomatik, Universitätsklinikum Hamburg-Eppendorf, Hamburg-Eppendorf, Deutschland; 3 Institut und Poliklinik für Medizinische Psychologie, Universitätsklinikum Hamburg-Eppendorf, Hamburg, Deutschland; 4 Professur für Erziehungswissenschaft mit dem Schwerpunkt Förderpädagogik („Emotionale und soziale Entwicklung“), Universität Siegen, Siegen, Deutschland

**Keywords:** Isolation, Wohlbefinden, Coronavirus, Social Distancing, Soziale Unterstützung, Isolation, Wellbeing, Coronavirus, Social distancing, Social support

## Abstract

**Hintergrund:**

Zur Eindämmung der COVID-19-Pandemie („coronavirus disease 2019“) wurden im Frühjahr 2020 Ausgang und Kontakte in Deutschland stark beschränkt. Studien weltweit lassen die Vermutung zu, dass die krisenbedingt angeordnete soziale Isolierung das Wohlbefinden der betroffenen Menschen signifikant beeinflusst. Um die gesundheitlichen Konsequenzen des Lockdowns verstehen und diesen präventiv begegnen zu können, wurde die gesundheitsbezogene Lebensqualität (gLQ) im Zusammenhang mit der Wohnsituation und dem subjektiv wahrgenommenen Haushaltsklima in diesem Zeitraum untersucht.

**Methodik:**

Eine durch vier Strategien deutschlandweit rekrutierte Stichprobe von *n* = 541 Erwachsenen (MW = 34 Jahre; 67 % weiblich) wurde mit standardisierten Instrumenten zu der gLQ und dem Haushaltsklima während des Lockdowns mittels eines Online-Surveys befragt.

**Ergebnisse:**

In der Stichprobe wurde im Mittel eine als mäßig einzustufende gLQ während des Lockdowns gefunden, die signifikant durch das subjektiv wahrgenommene Haushaltsklima vorhersagt wurde (*p* < 0,001). Alleinlebende Teilnehmende meldeten eine signifikant niedrigere gLQ zurück als Teilnehmende, die mit anderen Menschen zusammen in einem Haushalt lebten. Das Haushaltsklima sagte 26 % der Varianz der gLQ vorher; soziodemographische Merkmale klärten einen zusätzlichen Varianzanteil von 5 % auf.

**Diskussion:**

Die Hinweise dafür, dass ein besser eingeschätztes Haushaltsklima mit einer höheren gLQ während des Lockdowns zusammenhing, betonen die Bedeutung des häuslichen Umfelds. Bei zukünftigen Maßnahmen zur Pandemieeindämmung, die soziale Kontakte einschränken, sollten in der Bevölkerung das Bewusstsein für den Zusammenhang zwischen Wohlbefinden und Haushaltsklima erhöht werden sowie Hilfen für Menschen mit einem konfliktbelasteten häuslichen Umfeld zugänglich bleiben.

## Abstract (en)

**Background:**

To contain the coronavirus disease 2019 (COVID-19) pandemic, between March and May 2020 the stay-at-home order and contacts of people in Germany were severely restricted. Studies worldwide allow the assumption that social isolation ordered by the crisis can have a significant influence on the well-being of those affected. To better understand the health consequences of the COVID-19 lockdown and to be able to counteract them preventively, the health-related quality of life (HRQoL) of adults living in Germany was investigated in connection with the housing situation and the subjectively perceived household environment during this period.

**Methods:**

A sample of *n* = 541 adults (M = 34 years; 67% female), recruited nationwide by four different strategies, was interviewed with standardized instruments on HRQoL and household environment during the lockdown using an online survey.

**Results:**

In the investigated sample, an overall moderate HRQoL during the lockdown was found, which was significantly predicted by the subjectively perceived household environment (*p* < 0.001). Participants living alone reported significantly lower HRQoL than participants living with other people in the same household. The household environment predicted 26% of the variance in HRQoL; sociodemographic characteristics clarified an additional 5% variance.

**Conclusion:**

The evidence that a better perceived household environment was associated with higher HRQoL during the lockdown emphasizes the importance of the domestic environment during social isolation. Future pandemic containment measures that limit social contact should raise awareness of the link between well-being and household environment and ensure that assistance remains accessible to people with a conflict-ridden home environment.

## Hintergrund

Am 11.03.2020 erklärte die World Health Organization (WHO) das schwere Atemwegssyndrom „coronavirus disease 2019“ (COVID-19) zu einer Pandemie und veröffentlichte umfassende Maßnahmen zu deren Eindämmung [21]. In Deutschland begann am 23.03.2020 ein staatlich verordneter *Lockdown* mit Ausgangsbeschränkungen [4]. Der *Lockdown* umfasste das Schließen von Schulen, Universitäten, Gaststätten, Sportstudios und kulturellen Einrichtungen; nur systemrelevante Einrichtungen wie Supermärkte, Apotheken und Krankenhäuser blieben geöffnet. In der Öffentlichkeit durften sich Menschen während des *Lockdowns* nur allein, mit im gleichen Haushalt lebenden Personen oder mit einer weiteren nicht im gleichen Haushalt lebenden Person aufhalten [4]. Menschen aus Risikogruppen oder möglicherweise infizierte Personen kamen in eine freiwillige oder angeordnete häusliche Quarantäne. Die Bevölkerung wurde durch diese Regelungen aufgefordert, die Zahl an sozialen Kontakten so gering und konstant wie möglich zu halten. Außerdem sollte im Sinne des „*social distancing*“ an öffentlichen Orten ein Mindestabstand zu anderen Personen gehalten werden [4, 6, 11].

Hawton et al. [8] untersuchten in Großbritannien die gesundheitsbezogene Lebensqualität (gLQ) als mehrdimensionales Maß für das subjektive Wohlergehen [9, 14] im Kontext von sozialer Isolation im höheren Lebensalter. Bei 393 älteren Menschen (MW = 71,5 Jahre), die von sozialer Isolation betroffen oder bedroht waren, konnte ein negativer Zusammenhang zwischen der sozialen Isolation und der gLQ gefunden werden. In einer Stichprobe von 917 männlichen Seefahrern wurde ein positiver Zusammenhang zwischen der sozialen Unterstützung und der gLQ festgestellt [23]. Ein über soziale Medien verbreiteter Online-Survey zur gLQ während der COVID-19-Pandemie in China, in der 1139 Erwachsene im März 2020 teilnahmen, ergab keine signifikante Verringerung der gLQ in Folge von Ausgangsbeschränkungen, aber einen signifikanten Anstieg von Schmerzen, Ängsten oder Depressionen bei Teilnehmenden in einem höheren Lebensalter, mit chronischen Krankheiten, mit geringerem Einkommen oder mit der Sorge, an COVID-19 zu erkranken [13]. Eine Übersichtsarbeit, in der die Ergebnisse aus 24 international publizierten Studien zu den psychischen Folgen von Quarantänemaßnahmen zusammengefasst wurden, verwies auf erhöhte Raten von Depressionen, Schlaflosigkeit und psychische Belastungsreaktionen wie Wut, Stress und emotionale Erschöpfung, aber auch auf eine weniger starke Beeinträchtigung des Wohlbefindens bei freiwilliger im Vergleich zu angeordneter Quarantäne [3].

Auf der Grundlage dieser Studien lässt sich für den Zeitraum des COVID-19-Lockdowns ein Zusammenhang zwischen Haushaltsklima und gLQ vermuten. Angenommen wurde, dass Menschen, die während des Lockdowns im eigenen Haushalt soziale Unterstützung erhalten haben und das Haushaltsklima, als Maß für die soziale Unterstützung und Qualität der sozialen Beziehungen innerhalb ihres Haushalts [19], positiv bewerteten, eine höhere gLQ aufwiesen als Menschen, die allein lebten bzw. ein schlechtes Haushaltsklima erlebten.

## Methodik

Im Rahmen eines querschnittlich angelegten Online-Surveys wurden Erwachsene in Deutschland zur gLQ und zum Haushaltsklima während des Lockdowns befragt.

### Stichprobe

In die Studie wurden Erwachsene im erwerbstätigen Alter zwischen 18 und 67 Jahren eingeschlossen, die während des *Lockdowns* in Deutschland lebten. Die Rekrutierung der Teilnehmenden erfolgte über vier Rekrutierungsstrategien. Im ersten Schritt wurden Personen im universitären und sozialen Umfeld der AutorInnen zur Befragung eingeladen und 337 Teilnehmende rekrutiert. In der zweiten Phase wurde über Informationsgruppen zum Thema COVID-19 in sozialen Netzwerken, wie z. B. Facebook, zur Befragung eingeladen und 134 Teilnehmende gewonnen. Im dritten Schritt wurden Aushänge mit einem QR-Code, über den der Online-Fragebogen aufgerufen werden konnte, in systemrelevanten öffentlichen Orten wie Supermärkten in Hamburg verteilt und 38 Teilnehmende gewonnen. Eine 4. Woche, die genutzt wurde, um weitere Teilnehmende zu rekrutieren, diente der natürlichen, verselbständigten Verbreitung der Umfrage ohne aktive Ansprache, in der 59 Personen teilnahmen. Von der Gesamtstichprobe (*n* = 568) konnten nach dem Ausschluss von Personen, die nicht den Einschlusskriterien entsprachen und einer Datenbereinigung die Angaben von 541 Teilnehmenden (95,2 %) ausgewertet werden.

### Instrumente

Der Online-Fragebogen setzte sich aus Items zur standardisierten Erfassung von gLQ, Haushaltsklima und soziodemographischen Merkmalen zusammen. Um die gLQ zu erheben, wurde der 26 Items umfassende Fragebogen World Health Organization Quality of Life(WHOQOL)-BREF [22] genutzt (Beispielitem: „Wie gut können Sie Ihr Leben genießen?“). Die Items waren über 5‑stufige Likert-Skalen (z. B. 1 = „sehr unzufrieden“ bis 5 = „sehr zufrieden“) zu beantworten. Der WHOQOL-BREF umfasst entsprechend der vier Domänen der gLQ vier Skalen zum physischen, psychischen und sozialen Wohlbefinden sowie zur Umwelt der Teilnehmenden. Als zusätzliche Skala zur allgemeinen gLQ wurde der EUROHIS-QOL 8-item index aus 8 Items des WHOQOL-BREF herangezogen [18]. Aus dem Mittelwert der Scores in den 8 Items wurde eine Gesamt-QoL-Bewertung berechnet. Der Mittelwertscore wurde in einen standardisierten Score zwischen 20 und 100 umgewandelt, wobei ein höherer Score eine bessere Lebensqualität ausdrückt. Standardisierte Scores zwischen 20 und 60 können als geringe gLQ, Scores zwischen 61 und 80 als mäßige gLQ und Scores > 80 als hohe gLQ interpretiert werden.

Der Akzent des Konstrukts Haushaltsklima liegt auf dem subjektiven Erleben der häuslichen Umgebung. Das Haushaltsklima ist das perzipierte Klima, worunter nach Schneewind und Ruppert [20] die „atmosphärische“ Qualität der Beziehungen im eigenen Haushalt (S. 233) zu verstehen ist. Die Ermittlung des Haushaltsklimas erfolgte über vier Items, die angelehnt an die Definition von Schneewind et al. [19] entwickelt wurden und die Zustimmung zu Aussagen abfragten (Beispielitem: „Meine MitbewohnerInnen lassen mir den Freiraum, den ich benötige.“). Unter MitbewohnerInnen wurden alle Personen verstanden, mit denen die Teilnehmenden in einem Haushalt lebten (z. B. PartnerIn oder MitbewohnerIn in einer Wohngemeinschaft). Die Zustimmung zu den vier Aussagen wurde auf einer 5‑stufigen Likert-Skala (1 = „stimme überhaupt nicht zu“ bis 5 = „stimme voll und ganz zu“) erfasst und zu einer Skala zusammengefasst. Es konnten Summenscores zwischen 4 und 20 Punkten erreicht werden. Ein höherer Summenscore deutet auf ein besseres Haushaltsklima hin. Die Prüfung der internen Konsistenz ergab für die Skala ein als akzeptabel zu interpretierendes Cronbachs Alpha von 0,75 [2].

Als soziodemographische Merkmale wurden Alter, Geschlecht, Beziehungsstatus, Wohnsituation und Bildungsabschluss mit jeweils einem Item erfasst. Der Online-Fragebogen wurde mit dem Umfragetool Unipark [14] erstellt.

### Durchführung

Der Fragebogen wurde in einem Pretest mit 7 Teilnehmenden auf Praktikabilität und Expertenvalidität geprüft sowie hinsichtlich des Inhalts und Formats überarbeitet. Die Befragung begann am 05.05.2020 und endete am 01.06.2020. Die Teilnahme am Survey war freiwillig und anonym. Die Befragten konnten den Fragebogen jederzeit abbrechen oder Fragen überspringen. Die erste Seite des Online-Surveys enthielt die Studieninformationen. Um zu den Fragebogenitems zu gelangen, wurde das Einverständnis zur Teilnahme an der Befragung über den Einleitungstext abgefragt und durch die Teilnehmenden bestätigt. Die Teilnehmenden erhielten kein Incentive für die Beantwortung des Fragebogens, für die durchschnittlich 17 min benötigt wurden.

### Auswertung

Die Auswertung erfolgte über die Statistik- und Analysesoftware SPSS Version 27.0 (IBM Corporation, Armonk, NY, USA). Alle Daten wurden deskriptiv ausgewertet. Zur Beschreibung der Datenverteilung wurden absolute und relative Häufigkeit, Mittelwert (MW), Standardabweichung (SD) und Range angegeben. Die Pearson-Produkt-Moment-Korrelation wurde berechnet, um Zusammenhänge zwischen Haushaltsklima, der physischen, psychischen, sozialen, umweltbezogenen und allgemeinen gLQ sowie soziodemografischen Merkmalen zu ermitteln. Zur Analyse der Varianz der abhängigen Variable gLQ wurden mehrere multiple Regressionen mit der allgemeinen bzw. den vier Domänen der gLQ und der unabhängigen Variable Haushaltsklima als Modell 1 durchgeführt. In Modell 2 wurden jeweils die unabhängigen Variablen Alter, Geschlecht, Bildungsabschluss, Wohnsituation (alleinlebend vs. nicht alleinlebend) und Beziehungsstatus (Single vs. in Partnerschaft/verheiratet) hinzugefügt, um die zusätzliche Varianzaufklärung durch soziodemographische Merkmale für die gLQ zu berechnen.

## Ergebnisse

### Stichprobenbeschreibung

Die Teilnehmenden waren zwischen 18 und 67 Jahren alt. Das Durchschnittsalter lag bei 33,69 (SD = 13,38) Jahren. Insgesamt 67 % der Stichprobe waren weiblich; etwa 40 % gaben an, mit der Familie zusammen zu leben. Etwa 19 % (*n* = 105) der Teilnehmenden lebten während des *Lockdowns* allein. Über 40 % der Teilnehmenden verfügten über einen Universitäts- oder Hochschulabschluss, während 26 % das Abitur hatten. Insgesamt 66 % der Stichprobe gaben an, in einer Partnerschaft zu leben oder verheiratet zu sein (Tab. 1).

**Tab. 1:** Soziodemographie der Stichprobe (*n* = 541)

		MW (SD)
Durchschnittsalter (Jahre)		33,69 (13,81)
		% (*n*)
Anteil Frauen		66,7 (361)
Höchster Bildungsabschluss	Abitur	25,5 (138)
	Bachelor/Fachwirt	22,7 (123)
	Ausbildung	17,7 (96)
	Master/Diplom/Magister/Staatsexamen	17,2 (93)
	Mittlere Reife	7,6 (41)
	Fachhochschulreife	6,8 (37)
	Promotion/Habilitation	2,0 (11)
	Kein Abschluss	0,2 (1)
	Kein vergleichbarer Abschluss	0,2 (1)
Wohnsituation	Mit der Familie	39,6 (214)
	Mit dem/der PartnerIn	32,7 (177)
	Allein	19,4 (105)
	Wohngemeinschaft	7,6 (41)
	Sonstiges	0,7 (4)
Beziehungsstatus	In einer Partnerschaft	40,3 (218)
	Ledig	30,5 (165)
	Verheiratet	26,1 (141)
	Sonstiges	3,1 (17)

*MW* Mittelwert, *SD* Standardabweichung

### Gesundheitsbezogene Lebensqualität während des Lockdowns

Die gLQ während des *Lockdowns* wurde in der Gesamtstichprobe im Mittel mit einem Score von 71,96 bewertet (SD = 12,44), was einer mäßigen gLQ entspricht (Tab. 2). Das Minimum in der Bewertung der Domänen reichte von einem Score von 8,33 in der Skala soziale gLQ bis hin zu einem Score von 28,13 in der Skala Umwelt bzw. einem Minimum von 21,88 in der allgemeinen gLQ. Der höchste Mittelwert zeigte sich, außer in der allgemeinen gLQ, in der Skala Umwelt (MW = 76,51; SD = 13,37), während der niedrigste Mittelwert in der Skala soziale gLQ gefunden wurde (MW = 67,34; SD = 16,92). Männer wiesen eine signifikant höhere allgemeine gLQ auf (MW = 74,46; SD = 14,41) als Frauen (MW = 70,81; SD = 16,03), t = 3,00; *p* < 0,05. Alleinlebende Teilnehmende melden eine signifikant niedrigere gLQ (MW = 67,89; SD = 16,46) zurück als Teilnehmende, die mit anderen Menschen zusammen in einem Haushalt lebten (MW = 72,94; SD = 15,21), t = 3,00; *p* < 0,05.

**Tab. 2:** Gesundheitsbezogene Lebensqualität während des COVID-19-Lockdowns („coronavirus disease 2019“) allgemein und in den Domänen physisch, psychisch, sozial und umweltbezogen (*n* = 541)

		MW	SD	Range
gLQ	Allgemein Lebensqualität (EUROHISQOL)	71,96	15,58	21,88–100
	Umweltbezogen	76,51	13,37	28,13–100
	Physisch	75,57	15,06	17,86–100
	Psychisch	67,34	16,92	12,5–100
	Sozial	66,67	19,62	8,33–100

*gLQ* gesundheitsbezogene Lebensqualität, *MW* Mittelwert, *SD* Standardabweichung

### Haushaltsklima während des Lockdowns

Das Haushaltsklima während des *Lockdowns* wurde von den Teilnehmenden, die nicht allein lebten, im Durchschnitt als positiv bewertet (MW = 16,70; SD = 3,09). In dem Subsample der nicht alleinlebenden Personen bewerteten *n* = 436 Teilnehmende das Haushaltsklima mit Scores zwischen 5 und 20, die einem positiv eingeschätzten Haushaltsklima entsprechen. Etwa 20 % (*n* = 88) der nicht alleinlebenden Teilnehmenden bewerteten das Haushaltsklima mit einem Score von 20, der einem sehr positiven Haushaltsklima entspricht. Fast 90 % stimmten „eher“ oder „voll und ganz“ zu, während des *Lockdowns* den nötigen Freiraum von den MitbewohnerInnen zu bekommen (87,1 %; *n* = 380) sowie Hilfe und Unterstützung erhalten zu haben (85,6 %; *n* = 373). Etwa 86 % der nicht alleinlebenden Teilnehmenden stimmten „eher“ oder „voll und ganz“ zu, dass sie mit den MitbewohnerInnen über Gedanken, die sie beschäftigt haben, sprechen konnten. Knapp 20 % stimmten „eher“ oder „voll und ganz“ zu, dass zwischen ihnen und den MitbewohnerInnen während des *Lockdowns* eine angespanntere Stimmung herrschte als davor. Nicht alleinlebende Männer beurteilten das Haushaltsklima signifikant besser (MW = 17,17; SD = 2,53) als nicht alleinlebende Frauen (MW = 16,51; SD = 3,30); t = 11,33; *p* < 0,05.

### Zusammenhänge zwischen gLQ, Haushaltsklima und soziodemographischen Merkmalen

Zwischen dem Haushaltsklima und der allgemeinen gLQ (r = 0,51; *p* < 0,01) bzw. den vier Domänen der gLQ zeigten sich signifikante Zusammenhänge (Tab. 3). Alter (r = 0,18; *p* < 0,01) und Bildungsabschluss (r = 0,17; *p* < 0,01) der Teilnehmenden korrelierten signifikant positiv mit der allgemeinen gLQ, während sich eine signifikante negative schwache Korrelation zwischen Beziehungsstatus und allgemeiner gLQ zeigte (r = −0,14; *p* < 0,01). Das Haushaltsklima war mit keinem der untersuchten soziodemographischen Merkmale signifikant assoziiert.

**Tab. 3:** Pearson-Korrelationsmatrix für das Haushaltsklima, gesundheitsbezogene Lebensqualität und Soziodemographie (*n* = 541)

		1	2	3	4	5	6	7	8	9	10	11
1	Haushaltsklima	1	0,508^b^	0,383^b^	0,410^b^	0,410^b^	0,393^b^	0,078	−0,034	0,014	−0,076	0,066
2	Allgemeine gLQ	–	1	0,785^a^	0,810^a^	0,608^a^	0,735^a^	0,181^a^	0,170^a^	−0,013	−0,143^a^	0,093
3	Physische gLQ	–	–	1	0,674^a^	0,398^a^	0,598^a^	0,113^b^	0,115^b^	−0,011	−0,079	0,097^b^
4	Psychische gLQ	–	–	–	1	0,516^a^	0,557^a^	0,191^a^	0,094^b^	−0,041	−0,112^b^	0,132^a^
5	Soziale gLQ	–	–	–	–	1	0,398^a^	0,063	0,024	0,050	−0,309^a^	0,035
6	Umweltbezogene gLQ	–	–	–	–	–	1	0,125^b^	0,154^a^	−0,045	−0,035	0,046
7	Alter	–	–	–	–	–	–	1	0,229^a^	−0,072	−0,323^b^	−0,057
8	Bildungsabschluss	–	–	–	–	–	–	–	1	−0,064	−0,088	0,053
9	Wohnsituation	–	–	–	–	–	–	–	–	1	−0,069	0,014
10	Beziehungsstatus	–	–	–	–	–	–	–	–	–	1	0,057
11	Geschlecht	–	–	–	–	–	–	–	–	–	–	1

Wohnsituation: alleinlebend = 0, nicht alleinlebend = 1

Beziehungsstatus: Single = 0, in Partnerschaft/verheiratet = 1

*gLQ* gesundheitsbezogene Lebensqualität

^a^*p* < 0,001 (2-seitig)

^b^*p* < 0,05 (2-seitig)

### Varianzaufklärung der gLQ durch Haushaltsklima und soziodemographische Merkmale

Die lineare Regression zeigte für das Haushaltsklima eine starke signifikante Varianzaufklärung der allgemeinen gLQ (korrigiertes R^2^ = 0,26, Tab. 4). Der Bildungsabschluss konnte zusätzlich die allgemeine gLQ vorhersagen (Änderung in R^2^ = 0,05).

**Tab. 4:** Multiples Regressionsmodell zum Zusammenhang zwischen Haushaltsklima und gLQ während des COVID-19-Lockdowns („coronavirus disease 2019“; *n* = 541)

	Modell 1			Modell 2		
	B	β	*p*	B	β	*p*
Konstante	8,99	–	< 0,001	7,62	–	< 0,001
Haushaltsklima	0,40	0,51	< 0,001	0,40	0,50	< 0,001
Alter	–	–	–	0,02	0,09	0,053
Bildungsabschluss	–	–	–	0,25	0,16	< 0,001
Beziehungsstatus	–	–	–	−0,38	−0,06	0,14
*Modellanpassung Indizes*						
Korrigiertes R^2^	0,26			0,31		
F (df, df2), sig	F (1,433) = 150,36, *p* < 0,001			F (4,430) = 47,52, *p* < 0,001		
Änderung in R^2^	–			0,05		
Änderung in F2, sig	–			10,08, *p* < 0,001		

Abhängige Variable: allgemeine gLQ

Modell 1. Prädiktor: (Konstante), Haushaltsklima

Modell 2. Prädiktoren: (Konstante), Haushaltsklima, Alter, Abschluss, Beziehungsstatus

Beziehungsstatus: Single = 0, in Partnerschaft/verheiratet = 1

*gLQ *gesundheitsbezogene Lebensqualität

Das Haushaltsklima stellte eine moderate signifikante Varianzaufklärung der physischen (korrigiertes R^2^ = 0,15), psychischen (korrigiertes R^2^ = 0,17), sozialen (korrigiertes R^2^ = 0,17) und umweltbezogenen gLQ (korrigiertes R^2^ = 0,15) dar (Tab. 5 und 6). Für die physische gLQ konnte der Bildungsabschluss (β = 0,12 *p* < 0,02) als einzige Variable zusätzliche Varianzaufklärung liefern (Änderung in R^2^ = 0,02). Geschlecht und Alter erklärten signifikant zusätzlich 4 % der psychischen gLQ während des *Lockdowns* (korrigiertes R^2^ = 0,20). Die Varianz der Domäne soziale gLQ wurde um zusätzlich 8 % durch den Beziehungsstatus erklärt (korrigiertes R^2^ = 0,22). Die umweltbezogene gLQ wurde zusätzlich durch die Variable Bildungsabschluss vorhergesagt (Änderung in R^2^ = 0,03).

**Tab. 5:** Multiples Regressionsmodell zum Zusammenhang zwischen Haushaltsklima und den Domänen physische, psychische, soziale und umweltbezogene gesundheitsbezogene Lebensqualität (gLQ) während des COVID-19-Lockdowns („coronavirus disease 2019“, *n* = 541)

**Physische gLQ**						
	**Modell 1**			**Modell 2**		
	*B*	*β*	*p*	*B*	*β*	*p*
Konstante	45,12	–	< 0,001	36,49	–	< 0,001
Haushaltsklima	1,85	0,38	< 0,001	1,82	0,40	< 0,001
Alter	–	–	–	0,07	0,05	0,167
Bildungsabschluss	–	–	–	1,05	0,12	0,016
Beziehungsstatus	–	–	–	–	–	–
Geschlecht	–	–	–	1,96	0,07	0,116
**Modellanpassung Indizes**						
Korrigiertes R^2^	0,15			0,16		
F (df1, df2), sig	F (1,433) = 74,38; *p* < 0,001			F (4,430) = 22,13; *p* < 0,001		
Änderung in R^2^	–			0,02		
Änderung in F2, sig	–			4,17; *p* = 0,006		
**Soziale gLQ**						
	**Modell 1**			**Modell 2**		
	*B*	*β*	*p*	*B*	*β*	*p*
Konstante	25,97	–	< 0,001	30,91	–	< 0,01
Haushaltsklima	254	0,41	< 0,001	2,41	0,39	< 0,001
Alter	–	–	–	–	–	–
Abschluss	–	–	–	–	–	–
Beziehungsstatus	–	–	–	−13,14	−0,28	< 0,001
**Modellanpassung Indizes**						
Korrigiertes R^2^	0,17			0,24		
F (df1, df2), sig	F (1,433) = 87,32; *p* < 0,001			F (2,432) = 70,27; *p* < 0,001		
Änderung in R^2^	–			0,08		
Änderung in F2, sig	–			44,46; *p* < 0,001		

Anmerkungen: abhängige Variable: physische, psychische, soziale und umweltbezogene gLQ

Modell 1. Prädiktor: (Konstante), Haushaltsklima

Modell 2. Prädiktoren: (Konstante), Haushaltsklima, Alter, Abschluss, Wohnsituation, Beziehungsstatus

Wohnsituation: alleinlebend = 0, nicht alleinlebend = 2

Beziehungsstatus: Single = 0, in Partnerschaft/verheiratet = 1

Geschlecht: weiblich = 0, männlich = 1

*gLQ *gesundheitsbezogene Lebensqualität

**Tab. 6:** Multiples Regressionsmodell zum Zusammenhang zwischen Haushaltsklima und den Domänen physische, psychische, soziale und umweltbezogene gesundheitsbezogene Lebensqualität (gLQ) während des COVID-19-Lockdowns („coronavirus disease 2019“, *n* = 541)

**Psychische gLQ**						
	**Modell 1**			**Modell 2**		
	*B*	*β*	*p*	*B*	*β*	*p*
Konstante	30,50	–	< 0,001	19,08	–	< 0,001
Haushaltsklima	2,24	0,24	< 0,001	2,14	0,39	< 0,001
Alter	–	–	–	0,17	0,139	0,003
Bildungsabschluss	–	–	–	0,72	0,07	0,135
Beziehungsstatus	–	–	–	−1,56	−0,038	0,408
Geschlecht	–	–	–	3,59	0,11	0,009
*Modellanpassung Indizes*						
Korrigiertes R^2^	0,17			0,20		
F (df1, df2), sig	F (1,433) = 87,43; *p* < 0,001			F (5,429) = 23,10; *p* < 0,001		
Änderung in R^2^	–			0,04		
Änderung in F2, sig	–			6,00; *p* < 0,001		
**Umweltbezogene gLQ**						
	**Modell 1**			**Modell 2**		
	*B*	*β*	*p*	*B*	*β*	*p*
Konstante	49,31	–	< 0,001	42,11	–	< 0,001
Haushaltsklima	1,68	0,39	< 0,001	1,69	0,39	< 0,001
Alter	–	–	–	0,06	0,06	0,190
Abschluss	–	–	–	1,31	0,15	0,001
Beziehungsstatus	–	–	–	−0,45	−0,03	0,10
*Modellanpassung Indizes*						
Korrigiertes R^2^	0,15			0,18		
F (df1, df2), sig	F (1,433) = 78,94; *p* < 0,001			F (3,431) = 32,69; *p* < 0,001		
Änderung in R^2^	–			0,03		
Änderung in F2, sig	–			8,24; *p* < 0,001		

Anmerkungen: abhängige Variable: physische, psychische, soziale und umweltbezogene gLQ

Modell 1. Prädiktor: (Konstante), Haushaltsklima

Modell 2. Prädiktoren: (Konstante), Haushaltsklima, Alter, Abschluss, Wohnsituation, Beziehungsstatus

Wohnsituation: alleinlebend = 0, nicht alleinlebend = 2

Beziehungsstatus: Single = 0, in Partnerschaft/verheiratet = 1

Geschlecht: weiblich = 0, männlich = 1

*gLQ* gesundheitsbezogene Lebensqualität

## Diskussion

Die Studie zeigte, das Teilnehmende, die mit anderen Personen zusammenlebten bzw. ein positives Haushaltsklima mit den MitbewohnerInnen erlebten, eine signifikant höhere gLQ aufwiesen als alleinlebende Menschen bzw. Menschen mit einem negativ eingeschätztem Haushaltsklima. Das Haushaltsklima erklärte signifikant einen moderaten bis starken Varianzanteil an der allgemeinen gLQ sowie an der physischen, psychischen, sozialen und umweltbezogenen gLQ. Angesichts der Befunde von Xiao et al. [23] kann eine mögliche Erklärung für den Zusammenhang zwischen Wohnsituation bzw. Haushaltsklima und gLQ sein, dass Menschen in Zeiten von sozialer Isolation, wie dem *Lockdown* im Frühjahr 2020, soziale Unterstützung von Angehörigen im Haushalt benötigen [16]. Ein positives Haushaltsklima könnte z. B. funktionale Strategien im Umgang mit dem pandemiebedingten Stress gefördert und so die gLQ positiv beeinflusst haben [17].

Der Bildungsabschluss wurde als zusätzlicher signifikanter Prädiktor für die gLQ während des Lockdowns gefunden. Das Bildungsniveau zeigte sich bereits in anderen Untersuchungen in Deutschland als signifikanter Einflussfaktor für die gLQ, wobei zwischen Bildungsniveau und Wohlbefinden übereinstimmend ein positiver Zusammenhang gezeigt werden konnte [1, 12]. Die Metaanalyse von Furnée et al. [7], in der die Ergebnisse aus 40 Studien zum Einfluss des Bildungsniveaus auf die Gesundheit zusammengefasst wurden, ergab, dass mit jedem Bildungsjahr ein zusätzlicher positiver Effekt auf die Gesundheit und das Wohlbefinden angenommen werden kann.

Bei der Interpretation der Studienergebnisse müssen methodische Limitationen berücksichtigt werden. Die querschnittlich angelegte Online-Studie lässt keine Vorher-Nachher-Vergleiche für die Zeit vor dem Lockdown zu. Der Lockdown wurde im Zeitraum der Befragung schrittweise gelockert. Berücksichtigt werden muss zudem, dass sich der zur Befragung eingesetzte Fragebogen zur gLQ auf das Zeitfenster der vergangenen 2 Wochen vor dem Ausfüllen des Fragebogens bezieht. Je nach Befragungszeitpunkt und Wohnort waren die Ausgangsbeschränkungen aufgehoben und nicht systemrelevante Einrichtungen bereits wieder geöffnet, sodass das „normale“ Leben ohne Isolation teilweise wieder möglich war. Die unterschiedlich starke soziale Isolierung der Teilnehmenden während des Befragungszeitraums könnte daher Unterschiede bei den Bewertungen ihrer gLQ nach sich gezogen haben. Die vierstufige Rekrutierungsstrategie und Dokumentation des Stichprobenzuwachses zeigen dazu aber, dass die Teilnehmenden mehrheitlich noch während des deutschlandweit verordneten *Lockdowns* unter isolierten Bedingungen an der Befragung teilgenommen haben. Weitere Variablen, wie z. B. der sozioökonomische Status, körperliche oder psychische Vorerkrankungen, der Familienzusammenhalt oder die tatsächlich erhaltene soziale Unterstützung, die das Haushaltsklima und die gLQ als Ressourcen oder zusätzliche Belastungen beeinflusst haben könnten, wurden in der Studie nicht erhoben.

Nichtsdestotrotz lieferte die Befragung Hinweise dafür, dass das Zusammenleben mit Menschen und ein positiv erlebtes Haushaltsklima mit einer höheren gLQ während des Lockdowns zusammenhing. Die Ergebnisse der Studie unterstreichen die Bedeutung des häuslichen Umfelds während Phasen von krisenbedingter sozialer Isolierung. Bei zukünftigen Maßnahmen zur Pandemieeindämmung, die soziale Kontakte einschränken, sollten in der Bevölkerung das Bewusstsein für den Zusammenhang zwischen Wohlbefinden und Wohnsituation bzw. Haushaltsklima erhöht werden. Dazu sollten Hilfen für Menschen mit einem konfliktbelasteten häuslichen Umfeld (z. B. Konfliktberatung, Telefon- oder Online-Seelsorge, Jugendämter, Frauenhäuser) für Beratung und als Zufluchtsort zugänglich bleiben oder sogar verstärkt angeboten sowie Selbsthilfe- und Peer-Projekte initiiert werden. Kampagnen wie Mehr #Sicherheim für Frauen [5] und Aktion „Maske 19“ [10] bieten Opfern von häuslicher Gewalt gezielt während der COVID-19-Pandemie Unterstützung an und erhöhen das Bewusstsein für häusliche Gewalt in der Öffentlichkeit. Initiativen für unterstützende Netzwerke und Gemeinschaften sollten gefördert werden, um alleinlebende Menschen während der Zeit eines Lockdowns zu adressieren und sozial einzubinden.

## Fazit für die Praxis

- Die Bedeutung von sozialer Unterstützung für das Wohlbefinden wird während der sozialen Isolation zur Eindämmung der sowohl körperlich als auch psychisch bedrohlich wirkenden COVID-19-Pandemie („coronavirus disease 2019“) besonders deutlich.
- Das Zusammenleben mit anderen Menschen sowie ein positives Haushaltsklima scheinen in dieser Ausnahmesituation schützend zu wirken und zur psychischen Stabilisierung beizutragen.
- Allein oder unter belasteten häuslichen Bedingungen lebende Menschen können als besondere Risikogruppen für psychische Beeinträchtigungen während der Pandemie angenommen werden und sollten daher unter Berücksichtigung der geltenden Distanzregelungen durch niedrigschwellige und durchgehend erreichbare Angebote, die Austausch, (emotionale) Nähe, Unterstützung, Beratung oder Schutz ermöglichen, beispielsweise auch über die Nutzung von digitalen Medien gezielt adressiert werden.
- In der Forschung und Praxis sollten die sozialen und psychischen „Nebenwirkungen“ von schützenden Social-distancing-Maßnahmen als ein Anliegen der Prävention und Gesundheitsförderung, z. B. in Hinblick auf die langfristigen Auswirkungen der Pandemie mit wiederholten Lockdown-Phasen, Berücksichtigung finden.
